# Enhanced Multidimensional Luminescence Measurements Through Cyclodextrin Complexation

**DOI:** 10.6028/jres.093.109

**Published:** 1988-06-01

**Authors:** I. M. Warner, G. Nelson, G. Patonay, L. Blyshak, S. L. Neal

**Affiliations:** Department of Chemistry, Emory University, Atlanta, GA 30322

Cyclodextrins (CDxs) are cyclic oligosaccharides made up of 6, 7, or 8 glucopyranose units for *α*, *β*, and *γ*-CDx, respectively. These molecules are torus-shaped, with an interior cavity size ranging from 5 Å for the smaller *α*-CDx to 10 Å for the larger *γ*-CDx. While the CDx is water soluble, the cavity is characterized as hydrophobic. Consequently, an appropriately sized hydrophobic molecule can form an inclusion complex with the CDx. Often, the molecules involved in these complexes exhibit different properties than their uncomplexed forms, such as precipitation of the CDx complex, or a change in spectroscopic parameters of the included molecule. Cyclodextrin chemistry therefore offers a means for gaining selectivity and sensitivity in a variety of analytical measurements.

Many studies have demonstrated that the selectivity and accuracy of quantitative measurement of individual luminophors in a complex sample can be enhanced by simultaneous exploitation of multiple parameters. The combination of many multiple parameters could potentially provide specificity for the measurement. The most commonly used form of multidimensional luminescence measurement is the excitation-emission matrix (EEM), i.e., the measurement of the luminescence as a function of multiple excitation wavelengths (λ_ex_) and multiple emission wavelengths (λ_em_). In this talk, we discuss the use of CDxs to enhance selective detection via EEM measurements. Particular emphasis will be placed on the enhanced measurement of luminophors complexed with CDxs in the presence of select alcohols and quenchers. In addition, the use of solvent extraction and data analysis to reduce the dimensionality of the acquired EEM will be discussed.

Cyclodextrin complexation has been demonstrated to enhance the fluorescence of many types of included molecules. The lengthening of fluorescence lifetime [1], change in the fluorescence quantum yield [2], and shifts in the peak shape and position [3] have been observed as a consequence of complexation of fluorophors with CDxs. The addition of aliphatic alcohols to CDx systems has been shown to further enhance changes in these parameters [4]. These changes are often used to quantify the formation constants of a particular complex. Yet, in many instances, such changes are not of sufficient magnitude to quantify an analyte in a complex matrix.

One method of further enhancing the selectivity of CDx complexation is through the use of added quenchers. The accessability of some quenchers to a fluorophor is reduced by complexation of the fluorophor with CDxs. The presence of aliphatic alcohols further reduces the quenching of a CDx included fluorophor. A model system employing naphthalene and *β*-CDx will be discussed to demon-strate these principles. Appropriate Stern-Volmer type equations will be presented to explain the quenching phenomena observed in these studied.

Quenching may be successfully exploited for the analysis of a mixture of fluorescent molecules in the presence of CDx, where complexes of differing formation constants and protection from quenching interactions are present. In many cases, quenchers interact similarly with various classes of fluorescent molecules. Even the coupling of selective fluorescence measurements employing EEMs with quenching may not be sufficient to analyze a particular component. Quenching and CDx complexation offers a means of selectively analyzing a single component in a complex matrix. Figure 1 demonstrates the selective enhancement of pyrene in a five component mixture. These measurements will be discussed using fluorescence EEMs to demonstrate their utility in mixture analysis.

Cyclodextrin chemistry may also be successfully utilized in data reduction schemes for mixtures. Rank Annihilation schemes require the knowledge of one of the component spectra in order to elucidate other components present. Such a *priori* knowledge may not be available for some mixtures. The use of CDxs and quenchers can produce a single- or dual-component system from a more complex mixture, which can then be used as model component spectra. This type of data analysis scheme will be presented and discussed.

Research in the area of CDx extraction presently employs low solvent volumes and requires precipitation of the complex from aqueous solution. Analyses are performed after removal of the included compound from the CDx cavity by shaking the solid complex with another organic solvent. We discuss the development of a solvent extraction scheme for polynuclear aromatic hydrocarbons (PAHs) which allows for isolation of individual components from mixtures. In conventional solvent extraction, a solute is partitioned between two immiscible solvents. In this study, CDxs are used as aqueous phase components to increase the solubility of PAHs in water and thus increase their aqueous extraction efficiencies. The stereoselectivity and hydrophobicity of the CDxs allows for some degree of selective extraction based on the formation constants between different PAHs and the particular CDx used. Thus, CDxs can discriminate between organic phase solutes in an extraction scheme. For example, aqueous *γ*-CDx extracts only perylene from an organic perylene-anthracene mixture.

## Figures and Tables

**Figure 1:**
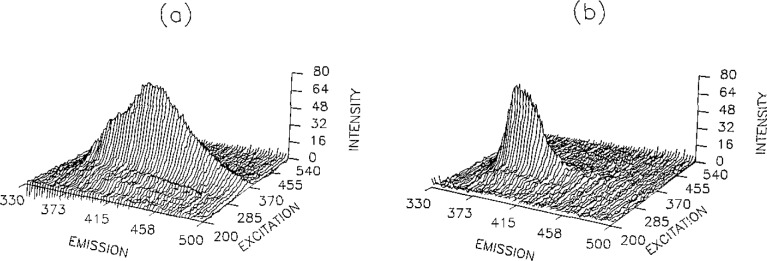
(a) EEM of five component mixture of acridine, 7,8-benzoquinoline, carbazole, fluoranthene, and pyrene, (b) Previous mixture in the presence of potassium iodide, 1*% tert*-butyl alcohol, and 1 millimolar *β*-CDx.
